# Draft genomes of two *Ruegeria* spp. isolated from the coral species *Pachyseris speciosa* in Singapore

**DOI:** 10.1128/mra.01303-24

**Published:** 2025-03-20

**Authors:** Joao Paulo Andre Pereyra, Aaron An Rong Loh, Dalong Hu, Lindsey Kane Deignan, Stephen Summers, Rebecca Josephine Case

**Affiliations:** 1 Singapore Centre for Environmental Life Sciences Engineering (SCELSE), Nanyang Technological University (NTU) 54761, Singapore, Singapore; 2 School of Biological Sciences, Nanyang Technological University 219572, Singapore, Singapore; 3 Singapore Centre for Environmental Life Sciences Engineering (SCELSE), National University of Singapore 37580, Singapore, Singapore; 4 Saw Swee Hock School of Public Health, National University Singapore 203377 , Singapore, Singapore; 5 St John’s Island National Marine Laboratory c/o Tropical Marine Science Institute, National University of Singapore (NUS) 541853, Singapore, Singapore; SUNY College of Environmental Science and Forestry 14797, Syracuse, New York, USA

**Keywords:** corals, marine microbiology, *Ruegeria*

## Abstract

Two *Ruegeria* strains, SCP10 (JBDZYF000000000) and SCP11 (JBDZYG000000000), were isolated from coral tissue and skeletal macerates of the resilient coral *Pachyseris speciosa* in Singapore. We present the genomes of these isolates, which contain genes involved in dimethylsulfoniopropionate metabolism and denitrification, good attributes for candidate coral probiotics.

## ANNOUNCEMENT

The genus *Ruegeria* is a member of the *Roseobacteraceae* family (1), an important marine group previously known as the marine Roseobacter clade (2). Species of *Ruegeria* play a role in marine carbon and sulfur cycling, through the catabolism of dimethylsulfoniopropionate (DMSP), the precursor for the climactically active gas dimethylsulfide (DMS) (3). They can be coral-associated, capable of protecting corals from bleaching through growth inhibition of coral pathogens (4), and forming mutualistic relationships within the coral holobiont to enhance health and survivorship (5), making *Ruegeria* strong probiotic candidates to enhance coral resilience.

Samples of *Pachyseris speciosa* were collected from the seawall at the northern side of Kusu Island, Singapore, and maintained in an outdoor aquaria tank with a constant seawater flow. Bacterial isolation methods were performed as described by Loh et al. (6). Briefly, coral samples were ground with a mortar and pestle, treated with proteinase K, vortexed with glass beads, and spread onto marine agar (Difco 2216) incubated at 33°C. SCP10 (for Singapore coral probiotic) was isolated from hydro-blasted coral tissue, and SCP11 was isolated from coral macerates (Table 1). Individual bacterial colonies were subcultured to ensure purity, and a single colony was used for each sample to inoculate marine broth (Difco 2216) and grown aerobically overnight for subsequent processing and sequencing.

**TABLE 1:** Genome features and GenBank accession numbers of the two *Ruegeria* spp. isolates

Isolate	Source	Genome size (bp)	No. of CDS^*a*^	No. of rRNAs	No. of tRNAs	G + C content (%)	No. of contigs	N_50_ (bp)	L_50_	Assembly accession no.	SRA accession no.	No. of reads	Average read length (bp)	Genome completeness (%)	Genome contamination (%)
SCP10	Tissue	4,483,924	4,396	3	49	56.9	111	476,927	3	JBDZYF000000000	SRR28745649	10,743,620	151	99.15	0.54
SCP11	Skeleton	5,119,499	4,882	3	52	56.8	82	285,744	7	JBDZYG000000000	SRR28745648	17,564,858	151	99.47	0.25

^*a*^ CDS, coding DNA sequences.

Genomic DNA was extracted with the DNeasy PowerSoil Pro Kit (Qiagen, Hilden, Germany). DNA library preparation and sequencing were performed by the SCELSE Sequencing Facility, NTU. DNA library preparation used the Accel-NGS 2S Plus Low Input DNA Library Prep Kit (Swift Biosciences, Ann Arbor, USA). Sequencing was performed using an Illumina HiSeq X Ten platform, version 2.5 (Illumina, San Diego, CA, USA), generating 150 bp paired-end reads.

Quality filtering and adaptor trimming were performed using Trimmomatic version 0.39 (7). Default parameters were used for all software unless specified. Genomes were assembled using the Shovill pipeline version 1.1.0 (https://github.com/tseemann/shovill) and the St. Petersburg Genome Assembler (SPAdes) version 3.15.5 (8). Annotations of the assembled genomes were carried out with Prokaryotic Genome Annotation Pipeline (PGAP) version 6.7 (9). The assemblies (Table 1) were checked for plasmid sequences with PlasmidFinder version 2.0.1 (10), with no plasmids found.

Isolates were identified using the Microbial Genomes Atlas (MiGA) (11) webserver, using the NCBI-Prok function, with the closest similarity of both genomes to *Ruegeria* sp. THAF33 (NZ_CP045384) based on average amino acid identity (AAI). Phylogenetic relatedness between each isolate and all publicly available *Ruegeria* reference genomes, as well as each other, was performed using the Orthologous Average Nucleotide Identity Tool, version 0.93.1 (12), and the Genome-To-Genome Distance Calculator, version 3.0 (13), to calculate the average nucleotide identity (ANI) and DNA-DNA hybridization (DDH) scores, respectively. Both genomes are <95% ANI and <70% DDH to each other and against all reference genomes, below the species threshold (14) (78.50% ANI and 80.78% AAI to each other).

Both genomes contained three different isoforms of DMSP lyases (*dddD, dddP,* and *dddQ*), which cleave DMSP to the cloud nucleating compound, DMS (15), as well as the anti-herbivory compound, acrylate (16). Analysis using GhostKOALA software version 2.0 (17) revealed the complete pathway for denitrification in SCP11, with the genomic potential to reduce nitrates and nitrites into dinitrogen (18).

## Data Availability

The SRA data were deposited to NCBI, and the complete genome sequences was deposited to GenBank under accession numbers listed in Table 1.
